# Congenital intrinsic proximal jejunal obstruction caused by a mucosal web near the duodenojejunal junction in a neonate: a case report and literature review

**DOI:** 10.1093/jscr/rjag257

**Published:** 2026-04-19

**Authors:** Semir Vranic, Asmir Jonuzi, Amira Mesic, Smail Halilovic, Jasmina Redzepagic, Zlatan Zvizdic

**Affiliations:** College of Medicine, QU Health, Qatar University, PO Box 2713, Doha, Qatar; Department of Pediatric Surgery, Clinical Center, University of Sarajevo, Bolnička 25, 71000 Sarajevo, Bosnia and Herzegovina; Department of Anesthesiology and Reanimation, Clinical Center, University of Sarajevo, Bolnička 25, 71000 Sarajevo, Bosnia and Herzegovina; Department of Pediatric Surgery, Clinical Center, University of Sarajevo, Bolnička 25, 71000 Sarajevo, Bosnia and Herzegovina; Department of Pathology, Clinical Center, University of Sarajevo, Bolnička 25, 71000 Sarajevo, Bosnia and Herzegovina; Department of Pediatric Surgery, Clinical Center, University of Sarajevo, Bolnička 25, 71000 Sarajevo, Bosnia and Herzegovina

**Keywords:** duodenojejunal junction, congenital obstruction, jejunal web, neonatal bilious vomiting

## Abstract

Congenital intrinsic obstruction at or near the duodenojejunal junction is exceptionally rare and most commonly results from incomplete embryonic recanalization, leading to the formation of a mucosal web. We report a 7-day-old term male neonate (birth weight 3350 g) who presented with persistent feeding intolerance and intermittent bilious vomiting since birth. Abdominal radiography showed marked dilation of the stomach and duodenum with distal bowel gas. An upper gastrointestinal contrast study revealed a conical narrowing at the duodenojejunal junction. Surgical exploration revealed a mucosal web located immediately distal to the duodenojejunal junction. Given the marked luminal disparity, simple web excision was deemed inadequate, and segmental resection with primary end-to-end jejunojejunal anastomosis was performed. Postoperative recovery was uneventful. Proximal jejunal webs near the duodenojejunal junction are rare but surgically correctable causes of neonatal bilious vomiting and should be considered in the differential diagnosis.

## Introduction

Bilious vomiting in neonates constitutes a surgical emergency and requires prompt evaluation. The differential diagnosis includes malrotation with midgut volvulus, Hirschsprung disease, sepsis, and a spectrum of congenital intrinsic or extrinsic intestinal obstructions. Type I atresia, first described by Louw and Barnard [1], is characterized by a mucosal web (also referred to as a mucosal diaphragm) that may result in complete or partial luminal obstruction. Congenital webs most commonly occur in the duodenum, where they typically present with the classic double-bubble sign. In contrast, fenestrated or partially obstructive webs may produce subtle or equivocal radiographic findings. Congenital intrinsic obstruction involving the duodenojejunal junction or proximal jejunum is exceedingly rare [2, 3]. It is also characterized by various clinical presentations. Owing to its atypical location and variable degree of obstruction, diagnosis can be challenging and requires a high index of suspicion combined with careful radiologic and intraoperative assessment. We present a rare case of a congenital mucosal web causing proximal jejunal obstruction located immediately distal to the duodenojejunal junction, highlighting key diagnostic considerations and operative strategies relevant to this unusual anatomical location.

## Case report

**Figure 1:**
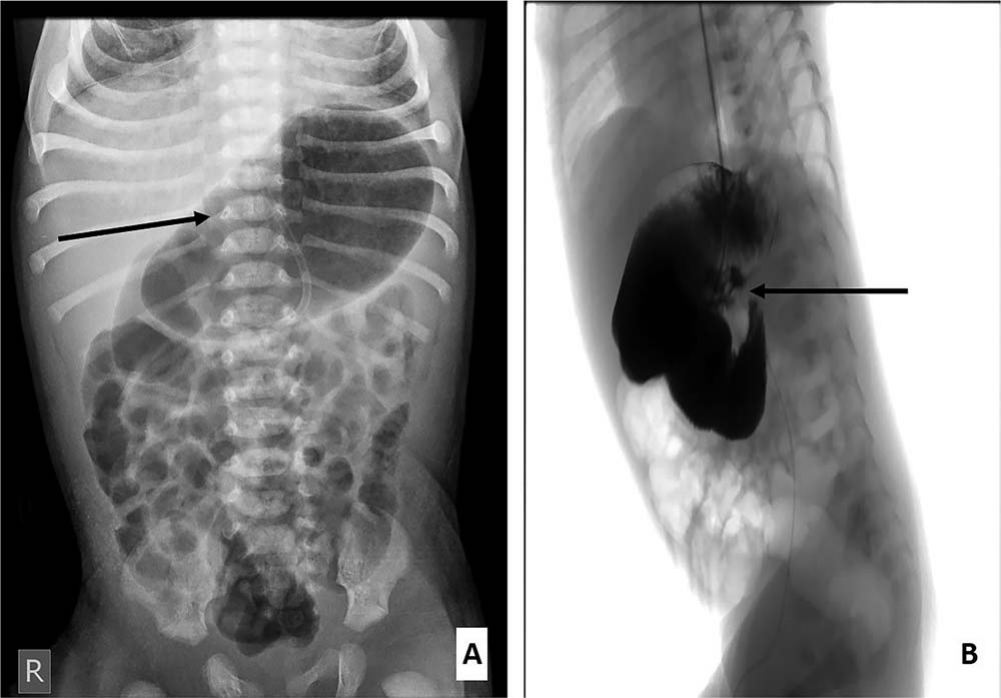
(A) Erect abdominal radiograph showing a markedly distended stomach (arrow) with aeration of the small and large intestines. (B) Contrast study demonstrating a markedly dilated stomach and duodenum with a conical narrowing at the duodenojejunal junction (labeled).

**Figure 2:**
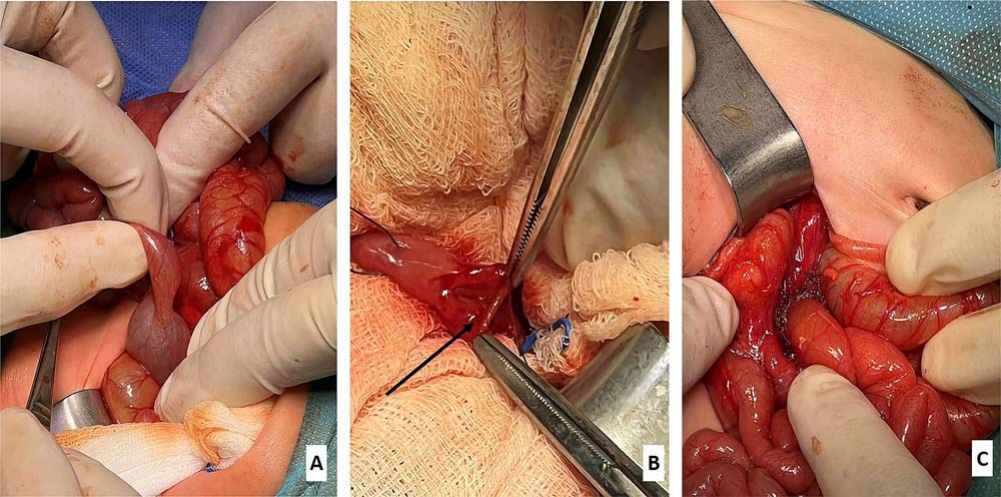
(A) Intraoperative view showing a grossly dilated stomach and duodenum with significant caliber disparity at the duodenojejunal junction. (B) Intraoperative identification of the mucosal web (labeled). (C) Following resection of the mucosal web, an end-to-end jejunojejunal anastomosis was performed distal to the duodenojejunal junction.

A 7-day-old term male neonate, delivered vaginally (birth weight 3350 g), presented with persistent feeding intolerance and intermittent bilious vomiting since birth. Prenatal ultrasound was unremarkable, and there was no family history of congenital anomalies. Erect abdominal radiography demonstrated marked dilatation of the stomach and duodenum with preserved distal bowel aeration (Fig. 1A). An upper gastrointestinal contrast study showed significant dilation of the stomach and all four portions of the duodenum, with a sharply tapered, conical narrowing at the duodenojejunal junction (Fig. 1B). The duodenojejunal junction was normally positioned to the left of the spine at the level of the pylorus, with normal passage of contrast into the distal small bowel, thereby excluding intestinal malrotation followed by a tension-free end-to-end jejunojejunal anastomosis (Fig. 2C). Exploratory laparotomy following Kocher’s maneuver revealed a grossly distended stomach and duodenum with pronounced caliber discrepancy at the duodenojejunal junction (Fig. 2A). Normal midgut rotation and fixation were confirmed, with no evidence of volvulus or Ladd’s bands. A mucosal web was identified in the proximal jejunum immediately distal to the duodenojejunal junction (Fig. 2B). Given the substantial luminal disparity, simple longitudinal enterotomy with web excision and transverse closure was avoided. Instead, segmental resection was performed, followed by a tension-free end-to-end jejuno-jejunal (Fig. 2C).

The postoperative course was uneventful. Bowel function returned promptly, enteral feeding was gradually initiated and advanced without difficulty, and the patient was discharged on postoperative Day 10. Follow-up at 3 and 6 months showed normal growth and feeding tolerance, with no gastrointestinal symptoms.

## Discussion

Intrinsic congenital obstruction of the proximal jejunum immediately distal to the duodenojejunal junction is extremely rare, with only a limited number of cases reported in the literature [2, 4]. These anomalies arise from incomplete embryologic recanalization and manifest as fenestrated or complete mucosal webs. Approximately 8% of all intestinal webs occur in the jejunum [4], and 30%–50% are associated with additional congenital anomalies [4, 5]; none were present in our patient. In the present case, the type I jejunal atresia was localized in the immediate post-duodenojejunal segment. While jejunal webs are uncommon overall, lesions in this proximal location may present either in the neonatal period or later in life with intermittent or nonspecific symptoms. To contextualize our findings, we compiled a focused review of reported pediatric cases of proximal/high type I jejunal atresia (defined as a mucosal web at the duodenojejunal junction or within 15 cm of the ligament of Treitz), emphasizing anatomic location, clinical presentation, surgical management, and outcomes (Table 1) [6–15]. As summarized in Table 1, these cases demonstrate heterogeneous clinical findings and variable operative strategies, underscoring the need for individualized surgical decision-making, as illustrated by our case. Clinical presentation largely depends on the degree of luminal obstruction. Complete webs typically present early with persistent bilious vomiting, whereas fenestrated webs may result in delayed or intermittent symptoms [10]. Radiologic evaluation may be inconclusive when distal bowel gas is present, highlighting the importance of correlating imaging findings with clinical suspicion. Standard management for duodenal webs consists of duodenotomy with web excision and transverse closure [2]. However, this approach may be suboptimal for lesions located at or just distal to the duodenojejunal junction, particularly in the presence of marked luminal discrepancy. In such cases, segmental resection with primary jejunojejunal anastomosis offers distinct advantages, including restoration of luminal continuity, reduced anastomotic tension, and a lower risk of postoperative stricture. This strategy was therefore appropriate for our patient and resulted in an excellent outcome.

**Table 1:** Reported pediatric cases/series of proximal/high type I jejunal atresia (mucosal web).

**Author (year)**	**Distance from Treitz/ DJ junction**	**Web type**	**Key imaging findings**	**Surgical procedure**	**Outcome**
Wong *et al*. (2024)	15 cm distal to the ligament of Treitz	Fenestrated	Partial proximal small bowel obstruction on contrast study	Segmental resection + end-to-end anastomosis	Good recovery; normal growth at 12 months
Diposarosa *et al*. (2024)	12 cm distal to the ligament of Treitz	Multiple webs (type I)	Severe proximal dilatation	Resection + anastomosis/web excision + jejunoileoplasty	Both patients died (POD 2 and POD 21)
Xiong *et al*. (2024)	≤10 cm from the ligament of Treitz (definition used)	Not specified	Not specified (registry-based cohort)	Predominantly resection + anastomosis	Higher NEC risk in the proximal group
Jeon *et al*. (2023)	DJ junction or ≤ 10 cm from Treitz	Type I in 3/7 cases	“Triple-bubble” sign on plain radiographs	Primary anastomosis	All tolerated oral feeds; no major complications
Khoei *et al*. (2021)	Proximal jejunum (distance not specified)	Fenestrated	CT: proximal jejunal obstruction	Web excision	Uneventful recovery
Abadi *et al*. (2021)	At the DJ junction/very proximal jejunum	Multiple webs	Upper GI obstruction	Web excision + jejunojejunostomy (Kimura)	Discharged without complications
Sharma *et al*. (2017)	Jejunum (distance not specified)	Fenestrated	Subacute obstruction; central web perforation	Web excision	Good postoperative course
Rajendran *et al*. (2014)	10 cm distal to the ligament of Treitz	Complete	Contrast study: upper jejunal obstruction	Resection + end-to-end anastomosis	Favorable recovery
Luo *et al*. (2010)	≤10 cm from the ligament of Treitz	Not specified	High small bowel obstruction	Duodenal derotation + tapering jejunoplasty + anastomosis	Feeding was tolerated within 14 days
Pandey *et al*. (2008)	~15 cm distal to the DJ flexure	Complete	Proximal bowel dilatation	Resection + end-to-end anastomosis	Uneventful recovery

DJ, duodenojejunal.

## Conclusion

In conclusion, congenital intrinsic proximal jejunal obstruction caused by a mucosal web near the duodenojejunal junction is a rare and diagnostically challenging condition. A careful radiologic evaluation, exclusion of malrotation, and meticulous intraoperative assessment are essential for accurate diagnosis. Segmental resection with primary anastomosis is a safe and effective surgical option in cases with significant luminal disparity. Early recognition and prompt surgical management are associated with excellent postoperative outcomes.
